# Perioperative Antifibrotic Treatment and Early Postoperative Outcomes in Patients With Idiopathic Pulmonary Fibrosis Undergoing Lung Cancer Surgery: A Systematic Review and Meta‐Analysis

**DOI:** 10.1111/1759-7714.70408

**Published:** 2026-10-25

**Authors:** Akimasa Sanagawa, Hideo Kato, Hajime Fujimoto, Tetsu Kobayashi, Takuya Iwamoto

**Affiliations:** ^1^ Department of Pharmacy Mie University Hospital Tsu Mie Japan; ^2^ Division of Clinical Medical Science, Department of Clinical Pharmaceutics Mie University Graduate School of Medicine Tsu Mie Japan; ^3^ Department of Pulmonary and Critical Care Medicine Mie University Faculty and Graduate School of Medicine Tsu Mie Japan

**Keywords:** antifibrotic therapy, idiopathic pulmonary fibrosis, lung cancer, pirfenidone, surgery

## Abstract

Patients with idiopathic pulmonary fibrosis (IPF) undergoing lung cancer surgery are at high risk of acute exacerbation of IPF (AE‐IPF), which is associated with high mortality. Although antifibrotic therapy (AFT) has been proposed as a preventive strategy, evidence remains limited and heterogeneous, particularly regarding early postoperative outcomes. We systematically searched major databases, including PubMed and Ichushi‐Web, through August 2025 to evaluate perioperative AFT in patients with IPF undergoing surgery. Eligible studies reported postoperative AE‐IPF or mortality within 30–90 days. Risk ratios (RRs) and 95% confidence intervals (CIs) were pooled using common‐ and random‐effects models. Five retrospective cohort studies predominantly evaluating pirfenidone were included. AFT was associated with a lower incidence of postoperative AE‐IPF both within 30 days (RR, 0.24; 95% CI, 0.07–0.76; *I* ^2^ = 0%) and 90 days (RR, 0.23; 95% CI, 0.09–0.57; *I* ^2^ = 0%). AFT was associated with lower mortality at 30 days (RR, 0.12; 95% CI, 0.02–0.91; *I* ^2^ = 0%) and 90 days (RR, 0.13; 95% CI, 0.02–0.76; *I* ^2^ = 0%). Sensitivity analyses, including a nationwide database study, supported the association, although the effect was attenuated. Evidence for nintedanib was limited to a small case series. Perioperative AFT, particularly with pirfenidone, was associated with lower rates of AE‐IPF and mortality, including within 30 days post‐lung cancer surgery. These findings underscore the clinical relevance of early postoperative outcomes and support further prospective studies to clarify the role of perioperative AFT.

## Graphical abstract

- Perioperative antifibrotic therapy (AFT), predominantly pirfenidone, was associated with a lower incidence of postoperative acute exacerbation of idiopathic pulmonary fibrosis (AE‐IPF) and mortality in patients undergoing lung cancer surgery.
- This systematic review and meta‐analysis separately evaluated early (30‐day) and later (90‐day) postoperative outcomes, demonstrating consistent associations across both time periods.
- These findings highlight the clinical relevance of early postoperative outcomes and support further randomized studies to clarify the role of AFT in the perioperative management of patients with IPF.

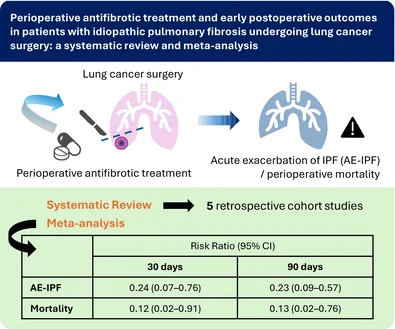

## 1 Introduction

Idiopathic pulmonary fibrosis (IPF) is a progressive, fibrosing interstitial lung disease characterized by an irreversible decline in lung function and a poor prognosis [1, 2]. In addition to its inherent morbidity, patients with IPF face a substantially increased risk of developing lung cancer, with cumulative incidence rates rising annually throughout the disease course [3]. Even after adjusting for age and smoking status, the incidence of lung cancer in patients with IPF is 5.0–8.3 times higher than in non‐IPF cases, suggesting that IPF creates a fertile ground for lung carcinogenesis [4].

Surgical resection, the standard treatment for early‐stage disease, presents a unique clinical dilemma when lung cancer coexists with IPF. Acute exacerbation of IPF (AE‐IPF) occurs in 9.3% of these cases and carries a mortality rate of 43.9% [5]. These events typically occur in the early postoperative period and are the leading cause of mortality within 30 postoperative days, underscoring the importance of short‐term outcomes [5].

The Japanese Association for Chest Surgery (JACS) score was developed as a predictive model for postoperative AE‐IPF to improve perioperative risk assessment [6]. This tool allows for patient stratification based on their risk of exacerbation. In addition, intraoperative and postoperative management strategies aim to minimize potential triggers of AE‐IPF, as tidal volumes and excessive fluid administration have been linked to an increased risk [7]. Furthermore, JACS‐guided risk assessment may influence surgical decision‐making, such as opting for less invasive procedures or avoiding surgery altogether. This could become even more crucial if effective pharmacological prophylaxis is established.

Regarding pharmacological prophylaxis, several agents, including sivelestat, corticosteroids, and ulinastatin, have been evaluated for perioperative administration, but none have demonstrated clinical efficacy [5]. More recently, retrospective studies have investigated antifibrotic agents such as pirfenidone and nintedanib, suggesting a potential benefit in reducing the incidence of postoperative AE‐IPF [8, 9, 10]. However, these findings have been limited to small observational cohorts or case series, and considerable heterogeneity remains in the timing of outcome assessment. While some studies have reported AE‐IPF and mortality at both 30 and 90 days postoperatively, others have assessed outcomes at only one of these time points, leading to a lack of uniformity across studies [8].

A recent systematic review and meta‐analysis synthesized the available data, suggesting that perioperative antifibrotic therapy (AFT) reduced the risk of AE‐IPF and mortality in patients with IPF undergoing lung cancer surgery [8]. However, this analysis primarily focused on 90‐day outcomes and did not adequately address early postoperative events. From a clinical perspective, outcomes within 30 days are highly relevant for both perioperative risk stratification and decision‐making, as they reflect the immediate impact of surgical stress and are often used as key endpoints in surgical trials. Indeed, an ongoing Phase III study has adopted the 30‐day AE‐IPF incidence as its primary endpoint, highlighting the growing recognition of early postoperative AE‐IPF as the most critical determinant of patient prognosis [11]. Presented at the European Society for Medical Oncology (ESMO) Congress 2024, this multicenter randomized trial did not demonstrate a statistically significant reduction in AE‐IPF with perioperative pirfenidone compared to control (6.1% vs. 10.3%, *p* = 0.339) [12]. While insufficient statistical power has been proposed as one possible explanation, other interpretations remain plausible, including differences in patient selection, perioperative management, and baseline risk profiles.

Given these gaps in the literature, a reappraisal of perioperative AFT is warranted, with careful attention to the timing of outcomes and its potential integration with surgical risk stratification. An updated systematic review and meta‐analysis could integrate existing evidence in a more clinically meaningful manner by differentiating between 30‐ and 90‐day outcomes, providing guidance for perioperative strategies in patients with lung cancer and concurrent IPF.

## 2 Methods

### 2.1 Search Strategy and Selection

This study was conducted according to the PRISMA Reporting Items for Systematic Reviews and Meta‐Analyses [13]. We systematically searched PubMed, Web of Science, CINAHL, Cochrane Library, ScienceDirect, and Ichushi‐Web databases for relevant articles published in English and Japanese up to August 6, 2025. The search terms used were: (“Idiopathic Pulmonary Fibrosis” OR “IPF”) AND (“Operative” OR “Surgery”) AND (“Anti‐fibrotic agents” OR “Pirfenidone” OR “Nintedanib”) AND (“Acute exacerbation” OR “Postoperative complications” OR “Mortality”).

### 2.2 Study Selection

Two investigators (A.S. and H.K.) independently screened the titles and abstracts of the retrieved articles. The full texts of eligible articles were then reviewed against the inclusion and exclusion criteria. Any disagreements were resolved by a third investigator (T.I.). Eligible studies met the following criteria: (i) randomized controlled trials (RCTs), retrospective observational studies, or cohort studies; (ii) involved patients with IPF; and (iii) investigated postoperative AE‐IPF and mortality.

In 2000, IPF was defined as a specific form of chronic fibrosing interstitial pneumonia of unknown cause, limited to the lungs, and associated with the histological appearance of typical interstitial pneumonia on surgical lung biopsy [14]. In 2011, the American Thoracic Society (ATS), European Respiratory Society (ERS), Japanese Respiratory Society (JRS), and Latin American Thoracic Society (ALAT) published guidelines for the diagnosis and management of IPF [15], which have since been periodically revised through international consensus [16, 17, 18].

### 2.3 Data Extraction and Risk‐of‐Bias Assessment

The following data were extracted from each study: authors, publication year, region, data source, study design and period, medications, controls, IPF diagnostic criteria, AE‐IPF definition, outcomes, confounder adjustment, patient population, demographic features, and surgical information. The Newcastle–Ottawa Quality Assessment Scale (NOS) was used to evaluate the quality of the included retrospective studies [19].

### 2.4 Definitions of AE‐IPF and Mortality

The primary outcome was the incidence of AE‐IPF within 30 days of surgery. Secondary outcomes included the incidence of AE‐IPF within 90 days and all‐cause mortality within 30 and 90 days. AE‐IPF was defined according to the AE‐IPF International Working Group [20].

### 2.5 Statistical Analyses

A meta‐analysis was conducted using EZR version 1.68 (Jichi Medical University, Tochigi, Japan) [21], a graphical user interface for R version 4.3.1 (The R Foundation for Statistical Computing, Vienna, Austria). EZR is a modified version of R Commander designed to incorporate statistical functions frequently used in biostatistics.

Statistical heterogeneity between studies was assessed using the *I*‐squared (*I* ^2^) measure. *I* ^2^ values were interpreted as follows: 0%–25%, low heterogeneity; 25%–50%, moderate heterogeneity; 50%–75%, substantial heterogeneity; 75%–100%, considerable heterogeneity. A *p* < 0.1 for Cochrane's *Q* test was considered indicative of statistically significant heterogeneity. However, given the small number of studies included in each meta‐analysis, both *I* ^2^ estimates and statistical tests of heterogeneity were interpreted cautiously due to their limited statistical power.

The fixed‐effects (common‐effect) model was applied when the data were homogeneous, and the random‐effects model was applied when heterogeneity was present. Unadjusted risk ratios (RRs) and 95% confidence intervals (CIs) were calculated for each retrospective cohort study. Pooled RRs and 95% CIs were calculated using a common‐effect model (Mantel–Haenszel method) and a random‐effects model (DerSimonian–Laird method), and the RRs from these models were compared. Given the small number of included studies, a formal assessment of publication bias was not performed. As a sensitivity analysis, outcomes including studies with zero events in one treatment arm were additionally analyzed using the Peto odds ratio (OR) method, which is appropriate for sparse event data and does not require continuity correction.

## 3 Results

### 3.1 Systematic Review

The study selection process is illustrated in Figure 1. Of 822 publications initially retrieved, 771 remained after duplicates were removed. Screening of titles and abstracts excluded irrelevant studies, leaving 12 potentially eligible publications. Two studies by Iwata et al. reported outcomes in overlapping patient populations [22, 23]. To avoid double counting, only the 2016 study, which featured a larger and more recent cohort, was included. Ultimately, five studies met the inclusion criteria and were incorporated into the final meta‐analysis.

**FIGURE 1:**
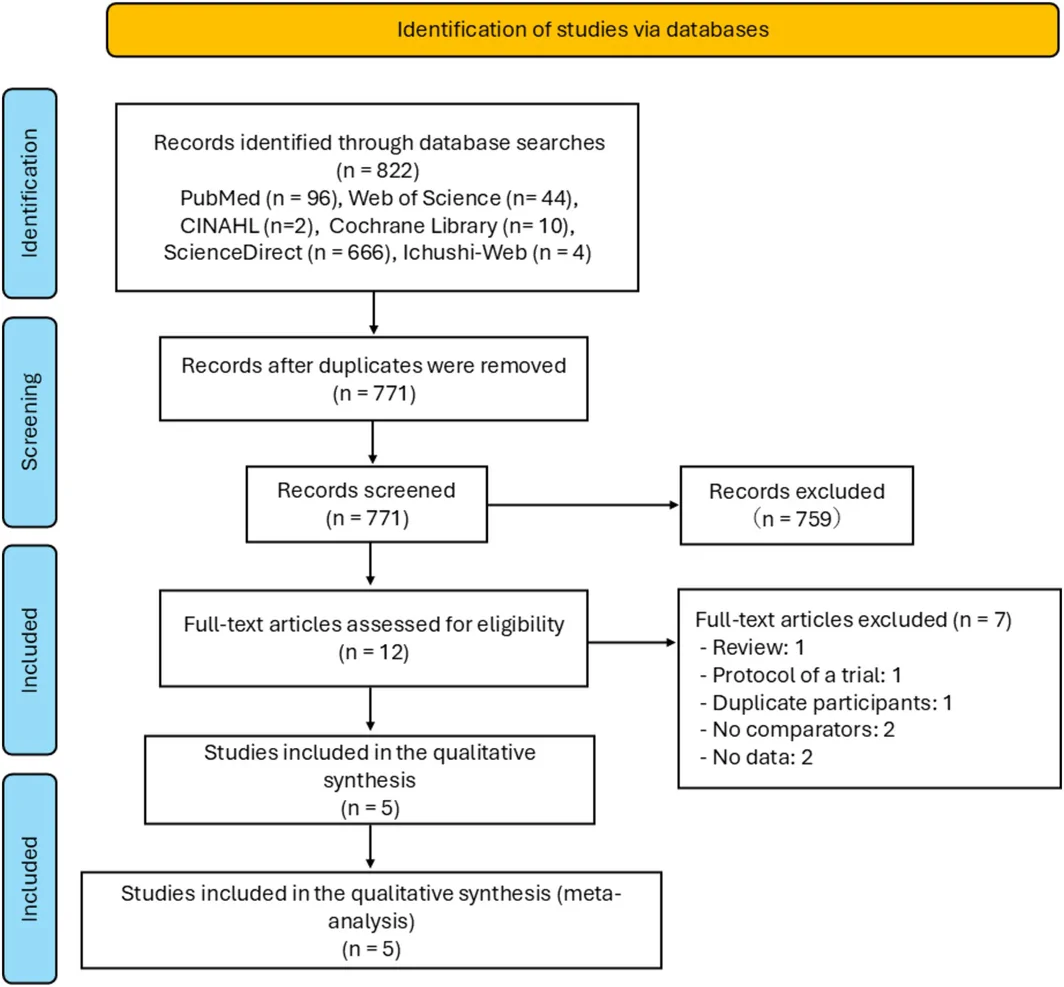
PRISMA flow diagram for the selection of eligible studies.

The characteristics of these studies are summarized in Table 1. The included studies were conducted in Japan [23, 24, 25, 26] and Italy [27], all focusing on patients with IPF. Consequently, the effects of perioperative AFT in patients with non‐IPF interstitial lung diseases were not evaluated. All publications were retrospective cohort studies with NOS scores ranging from 7 to 9, indicating moderate to high quality. In the Japanese studies, AFT exclusively consisted of pirfenidone [23, 24, 25, 26]. An Italian study included patients receiving chronic nintedanib therapy; however, pirfenidone accounted for 82.2% of the AFT group when considering both chronic and prophylactic use [27]. Prophylactic pirfenidone was typically initiated approximately 2–5 weeks before surgery with dose escalation. For chronic pirfenidone treatment, the timing of discontinuation before surgery was not specified. In contrast, nintedanib was stopped 2–5 days presurgery and restarted once the bleeding risk had resolved [27].

**TABLE 1:** Characteristics of studies included in the meta‐analysis.

Study	Design	Region	Data source	Period	Medications	Controls	IPF diagnosis	Postoperative AE‐IPF definition	Outcomes	Confounder adjustment	NOS score
Iwata et al. [23]	Retrospective cohort	Japan	Single institution	Oct 2006–Oct 2014	Pirfenidone (administered 2–5 weeks before the operation: starting at 600 mg/day for 1–2 weeks, increased to 1200 mg/day for 1–2 weeks, then increased to 1800 mg/day if possible)	Historical controls	Preoperative biopsy or HRCT (ATS/ERS/JRS/ALAT criteria 2011)	Subjective worsening of dyspnea, new bilateral radiologic opacities, no evidence of infection, exclusion of alternative causes	AE‐IPF (30‐ and 90‐day)	Perioperative pirfenidone treatment, JACS risk score	9
Kanayama et al. [24]	Retrospective cohort	Japan	Single institution	Jan 2011–Apr 2018	Pirfenidone (starting at 600 mg/day for 1–2 weeks, then increased to 1200 mg/day for 1–2 weeks)	Historical controls	HRCT and pathological findings (ATS/ERS/JRS/ALAT criteria 2011)	Intensified dyspnea, increased interstitial shadow on imaging, a decrease in arterial oxygen tension of > 10 mmHg, no evidence of infection, exclusion of alternative causes (ATS/ERS/JRS/ALAT criteria 2011)	AE‐IPF (30‐ and 90‐day)	Risk groups classified by JACS risk score	9
Sekihara et al. [25]	Retrospective cohort	Japan	Single institution	Jan 2011–Sep 2016	Pirfenidone (administered 2–4 weeks before the operation: starting at 600 mg/day for 1–2 weeks, then increased to 1200 mg/day for 1–2 weeks)	Historical controls	Evaluation and re‐categorization based on HRCT (ATS/ERS/JRS/ALAT criteria 2011)	Subjective worsening of dyspnea, new bilateral radiologic opacities, no evidence of infection, exclusion of alternative causes, a decrease in arterial oxygen tension of > 10 mmHg (ATS/ERS/JRS/ALAT criteria 2011)	AE‐IPF (30‐ and 90‐day), mortality (30‐ and 90‐day)	Specific adjustment not shown	7
Urushiyama et al. [26]	Retrospective cohort	Japan	National inpatient database	Jul 2010–Mar 2018	Pirfenidone (prescribed pirfenidone within 45 days before surgery)	Not prescribed pirfenidone within 45 days before surgery	The diagnosis of IPF is recorded in Japanese text	Mechanical ventilation under endotracheal intubation and/or intravenous infusion of a high‐dose corticosteroid within 30 days after surgery	30‐Day severe respiratory complications, 30‐day mechanical ventilation, 30‐day high‐dose steroid administration, 30‐day in‐hospital mortality	Age, sex, BMI, Barthel index, Hugh–Jones Dyspnea Scale, smoking history, previous admissions, surgery for lung cancer, surgery of other organs, patients with cancer, patients with COPD, patients with heart disease, oral corticosteroids before surgery, days from admission to surgery, blood transfusion during surgery, anesthesia time	9
Bongiolatti et al. [27]	Retrospective cohort	Italy	Multiple institutions	Jan 2015–Dec 2022	Pirfenidone (administered 2–4 weeks before the operation, starting at 801 mg/day, increased to 2403 mg/day for at least 7–10 other days) Nintedanib (stopped for 2–5 days before surgery, restarted when the bleeding risk was nullified)	Lack of use of perioperative AFT (pirfenidone or nintedanib)	Multidisciplinary discussion at an ILD Referral Center (ATS/ERS/JRS/ALAT criteria 2011 or ATS/ERS/JRS/ALAT Clinical Practice Guideline 2018)	Acute, clinically significant respiratory deterioration with new, widespread alveolar abnormality, excluding alternative etiologies, developed < 1 month after curative‐intent lung surgery for cancer in IPF patients	AE‐IPF (90‐day), mortality (30‐ and 90‐day)	Age, sex, FVC%, DLCO%, GAP index, AFT use	9

Abbreviations: AE, acute exacerbation; AFT, antifibrotic therapy; ALAT, Latin American Thoracic Society; ATS, American Thoracic Society; BMI, body mass index; COPD, chronic obstructive pulmonary disease; DLCO, diffusing capacity of the lung for carbon monoxide; ERS, European Respiratory Society; FVC, forced vital capacity; GAP index, gender, age, physiology index; HRCT, high‐resolution computed tomography; ILD, interstitial lung disease; IPF, idiopathic pulmonary fibrosis; JACS, Japanese Association for Chest Surgery; JRS, Japanese Respiratory Society.

Urushiyama et al. [26] utilized the Japanese national inpatient database to identify IPF patients undergoing surgery under general anesthesia. Patients were divided into preoperative pirfenidone and control groups, and propensity score matching was performed to assess pirfenidone's effects on AE‐IPF and mortality. Unlike the other studies, this study included cases beyond lung cancer surgery. Furthermore, AE‐IPF was not defined according to guideline criteria but rather as severe respiratory complications or the requirement for mechanical ventilation with endotracheal intubation or high‐dose steroids within 30 days post‐surgery. Therefore, this study was excluded from the main meta‐analysis and only considered for sensitivity analysis using 30‐day mortality data.

Regarding outcomes, 30‐day AE‐IPF incidence was reported by Iwata et al., Kanayama et al., and Sekihara et al. [23, 24, 25], while 90‐day AE‐IPF incidence was reported in studies by Kanayama et al., Sekihara et al., and Bongiolatti et al. [23, 24, 25, 27]. Thirty‐day mortality data were available from the studies by Sekihara et al., Urushiyama et al., and Bongiolatti et al. [25, 26, 27], and 90‐day mortality data were reported by Sekihara et al. and Bongiolatti et al. [25, 27].

Other outcomes varied substantially across the studies. None of the included studies compared the incidence of adverse events between the AFT and control groups, precluding a meta‐analysis of safety outcomes beyond AE‐IPF and mortality.

### 3.2 Meta‐Analysis

#### 3.2.1 Incidence of Postoperative AE‐IPF

A meta‐analysis demonstrated that AFT significantly reduced the incidence of postoperative AE‐IPF. Forest plots of AE‐IPF outcomes are presented in Figure 2. The pooled RR for 30‐day AE‐IPF was 0.24 (95% CI, 0.07–0.76; *I* ^2^ = 0%; Figure 2A), and for 90‐day AE‐IPF, it was 0.23 (95% CI, 0.09–0.57; *I* ^2^ = 0%; Figure 2B). Although *I* ^2^ was 0% in both analyses, the small number of included studies limits the ability to reliably detect between‐study heterogeneity. A sensitivity analysis using the Peto OR yielded similar results (overall OR, 0.25; 95% CI, 0.09–0.68; Figure S1).

**FIGURE 2:**
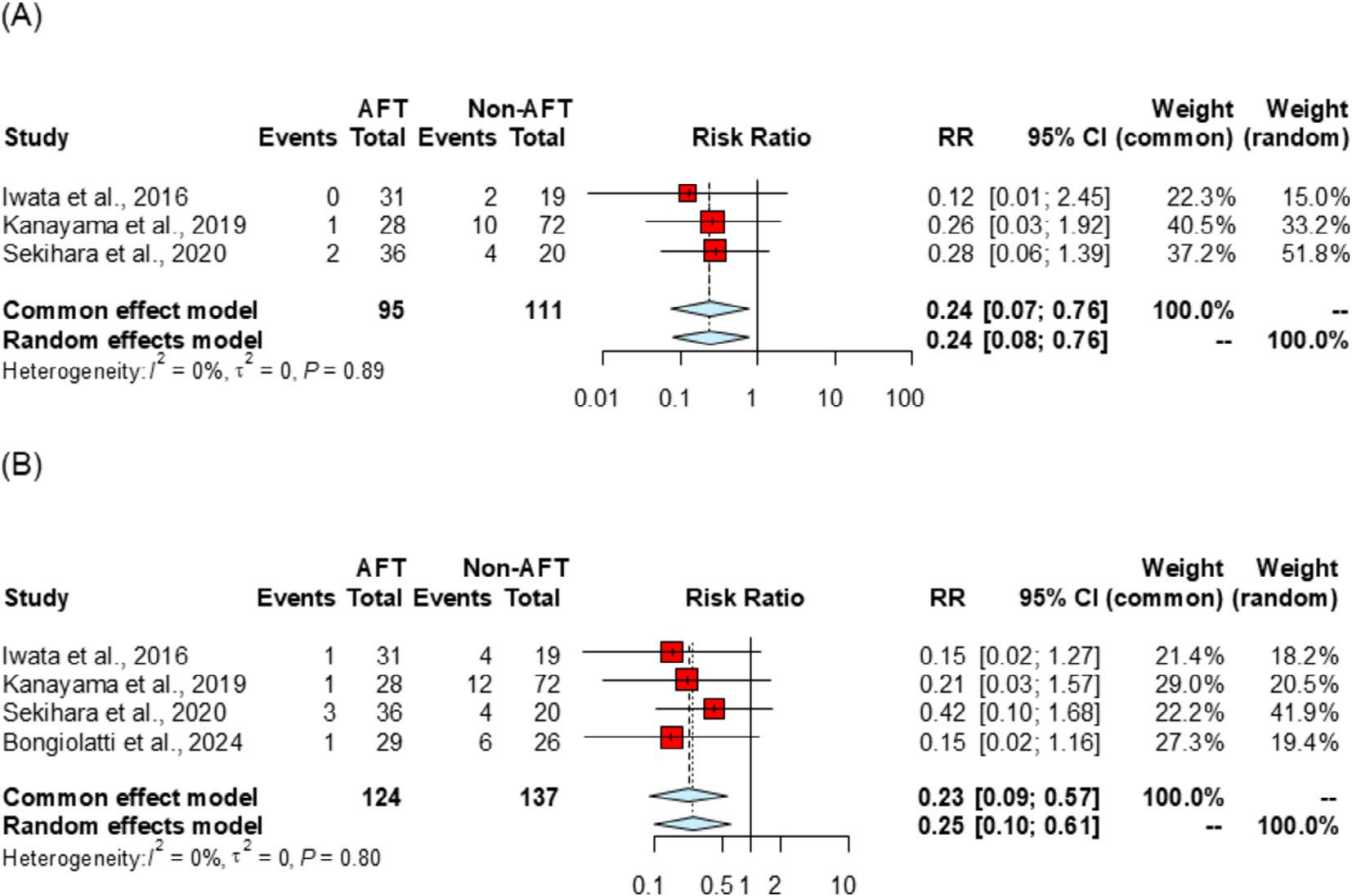
Forest plot of risk ratios for acute exacerbation of idiopathic pulmonary fibrosis (AE‐IPF) in patients with idiopathic pulmonary fibrosis undergoing lung cancer surgery, comparing those treated with antifibrotic therapy to those receiving non‐antifibrotic therapy. (A) 30‐Day AE‐IPF, (B) 90‐day AE‐IPF. AFT, antifibrotic therapy; CI, confidence interval; RR, risk ratio.

#### 3.2.2 Mortality

AFT is also associated with reduced postoperative mortality rates. Forest plots of mortality outcomes are shown in Figure 3. The pooled RR for 30‐day mortality was 0.12 (95% CI, 0.02–0.91; *I* ^2^ = 0%; Figure 3A), and for 90‐day mortality, it was 0.13 (95% CI, 0.02–0.76; *I* ^2^ = 0%; Figure 3B). Although no substantial heterogeneity was detected statistically, the small number of studies limits the power to reliably assess between‐study heterogeneity. Sensitivity analyses using the Peto OR also showed consistent findings for both 30‐day mortality (overall OR, 0.08; 95% CI, 0.01–0.47) and 90‐day mortality (overall OR, 0.12; 95% CI, 0.03–0.52) (Figures S2 and S3).

**FIGURE 3:**
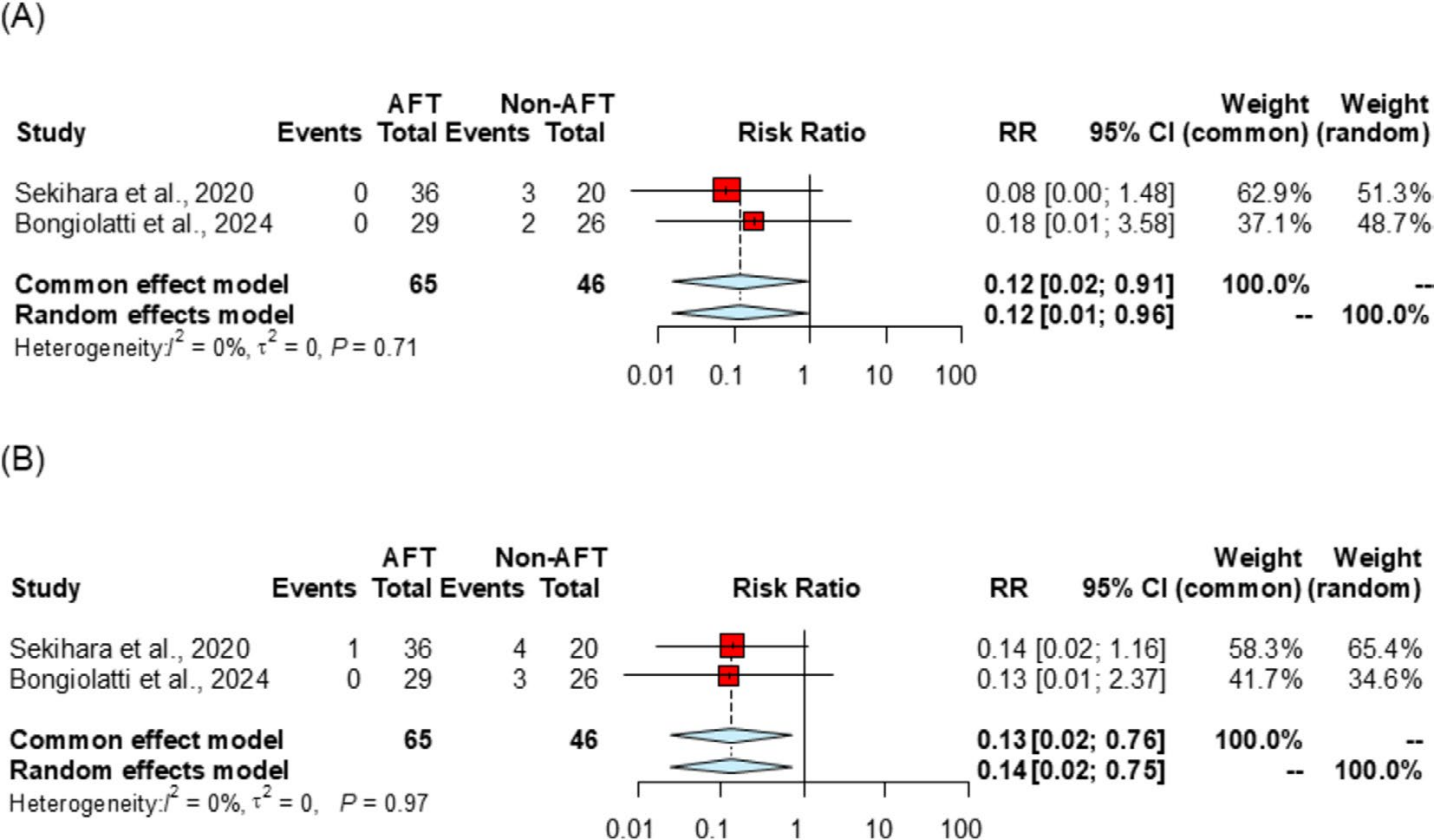
Forest plot of risk ratios for mortality in patients with idiopathic pulmonary fibrosis undergoing lung cancer surgery, comparing those treated with antifibrotic therapy to those receiving non‐antifibrotic therapy. (A) 30‐Day mortality, (B) 90‐day mortality. AFT, antifibrotic therapy; CI, confidence interval; RR, risk ratio.

#### 3.2.3 Subgroup and Sensitivity Analyses

Among the five eligible studies, four specifically evaluated pirfenidone, while one study included a small proportion of patients treated with nintedanib in addition to pirfenidone. Due to this imbalance, drug‐specific subgroup analyses could not be performed.

The study by Urushiyama et al. [26] differed from the other included studies in several important aspects: it was based on a nationwide inpatient database, enrolled patients undergoing a variety of surgeries beyond lung cancer resection, and defined AE‐IPF within 30 days after surgery rather than according to international consensus criteria. Detailed information on the underlying histological or radiological pattern of IPF was also unavailable. Therefore, this study was excluded from the meta‐analysis.

To assess the robustness of the findings, we performed a sensitivity analysis including the 30‐day mortality data from Urushiyama et al. [26]. In this analysis, the pooled RR for 30‐day mortality was 0.30 (95% CI, 0.10–0.92) under the common‐effect model. The corresponding random‐effects model produced a pooled RR of 0.31 (95% CI, 0.09–1.03). The results are shown in Figure 4. The direction of the association was unchanged after inclusion of the Urushiyama study, although statistical significance was attenuated under the random‐effects model.

**FIGURE 4:**
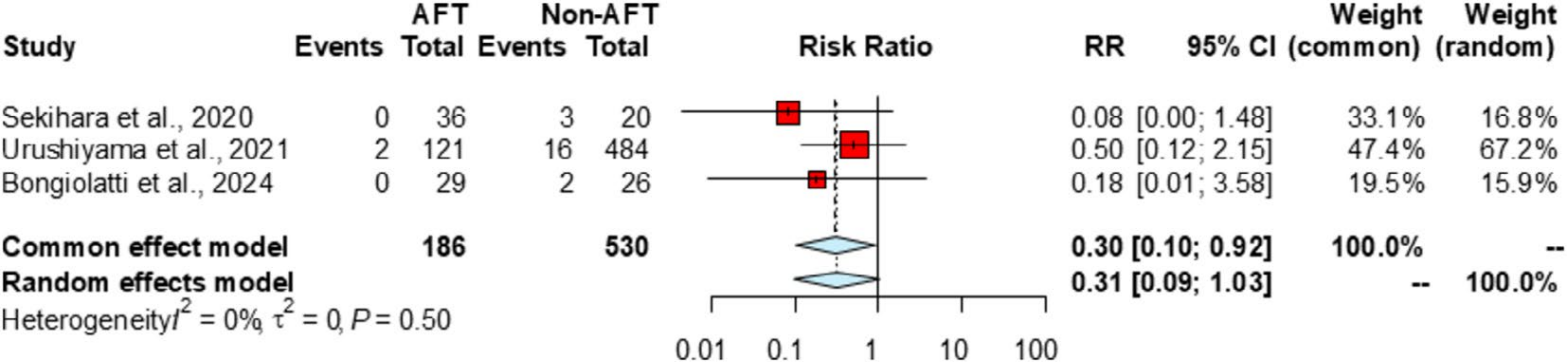
Sensitivity analysis of 30‐day mortality, including the Urushiyama study. AFT, antifibrotic therapy; CI, confidence interval; RR, risk ratio.

## 4 Discussion

In this systematic review and meta‐analysis, preoperative AFT was associated with a lower incidence of AE‐IPF and mortality not only within 90 days but also within 30 days after lung cancer surgery (RR for 30‐day AE‐IPF: 0.24; 95% CI, 0.07–0.76; RR for 30‐day mortality: 0.12; 95% CI, 0.02–0.91). Although *I* ^2^ was 0% across the primary meta‐analyses, the small number of included studies limits the ability to draw firm conclusions regarding between‐study heterogeneity. Our findings are partly consistent with those reported by Srivali et al. [8], who demonstrated an association between preoperative AFT and a lower 90‐day postoperative AE‐IPF and mortality. The present study extends these observations by demonstrating significant associations with both AE‐IPF and mortality 30 days after lung cancer surgery. However, these findings differ from those of the recently presented Phase 3 NEJ034 trial [12], which did not demonstrate a significant reduction in the incidence of 30‐day postoperative AE‐IPF with perioperative pirfenidone.

Several factors may explain the discrepancy between the observational studies included in the present meta‐analysis and the randomized evidence. First, all studies included in our meta‐analysis were retrospective observational studies, and treatment allocation was determined according to clinical practice rather than randomization. Consequently, confounding by indication cannot be excluded, as patients receiving perioperative pirfenidone may have differed from untreated patients with respect to baseline disease severity, pulmonary function, perioperative management, and other unmeasured clinical characteristics associated with postoperative outcomes. In addition, three studies used historical control groups, making it difficult to distinguish the effects of pirfenidone from temporal improvements in surgical techniques, perioperative management, and postoperative care [23, 24, 25]. These sources of bias partly explain the discrepancy between observational findings and the randomized evidence. Conversely, the currently available information regarding the NEJ034 trial is limited to a conference presentation, and detailed analyses of baseline characteristics, subgroup effects, protocol adherence, and secondary endpoints have not yet been fully reported. Therefore, publication of the full trial results is awaited to better understand the reasons why the primary endpoint was not achieved and to facilitate a more comprehensive comparison between randomized and observational evidence.

While insufficient statistical power has been proposed as one possible explanation, other factors, including differences in patient selection, perioperative management, and baseline risk profiles, may also contribute to the discrepancy between randomized and observational findings. Sensitivity analyses using the Peto OR for outcomes including single‐zero‐event studies yielded findings consistent with the primary analyses, suggesting that the observed associations were not materially influenced by the analytical approach used for sparse event data. However, the effect on 30‐day mortality was attenuated when non‐lung cancer surgeries were included, suggesting that the clinical benefit of lung cancer resection may be more pronounced. Notably, four of the five included studies specifically evaluated pirfenidone [23, 24, 25, 26], whereas the remaining study included patients receiving both pirfenidone and nintedanib [27]. Therefore, although the present meta‐analysis evaluated AFT as a treatment category, the available evidence was predominantly based on pirfenidone, and the independent contribution of nintedanib to the observed associations remains uncertain.

Regarding safety, three of the included studies provided detailed descriptions of adverse events in the AFT group, most of which were mild (Grades 1–2) and did not lead to postponement or cancellation of surgery [23, 24, 25]. Common events included nausea, photosensitivity, and rash, with occasional laboratory abnormalities such as elevated aminotransferase levels. No severe pirfenidone‐related complications (Grade 3) were observed, and most patients could restart pirfenidone soon after surgery. Comparative analyses in two studies [23, 24] demonstrated no significant differences in the incidence of postoperative complications between the AFT and non‐AFT groups. Similarly, Bongiolatti et al. [27] reported no significant difference in postoperative complications classified as Clavien–Dindo grade > 3a between the AFT and control groups. However, the included studies did not consistently report postoperative complications other than AE‐IPF according to specific postoperative time points. By contrast, Urushiyama et al. [26], although excluded from the main meta‐analysis due to differences in AE‐IPF definition, found that pirfenidone use was associated with a significantly lower risk of severe postoperative respiratory complications. Altogether, although the safety profile of pirfenidone appears to be acceptable in the perioperative setting, the absence of consistent comparative safety data across studies precludes a formal meta‐analysis of adverse events.

An RCT is currently underway [11]. The protocol included multiple secondary endpoints, such as a postoperative AE‐free period within 90 days, safety information on complications due to pirfenidone and surgery, 2‐year postoperative survival rate, recurrence‐free survival rate of lung cancer, progression‐free survival rate of IPF, and subgroup analyses based on the JACS score and imaging review. The forthcoming publication of the Phase 3 trial results, including detailed analyses of why the primary endpoint was not met at ESMO 2024 [12], is highly anticipated. This ongoing trial will provide crucial evidence to guide the optimal use of AFT, particularly pirfenidone, and to clarify its role in the prevention of postoperative AE‐IPF and early mortality.

No comparative studies evaluating the effect of nintedanib on postoperative AE‐IPF were identified, precluding drug‐specific subgroup analysis. A small case series of 12 patients with IPF who received nintedanib before lung cancer surgery has been reported [9]. In this series, nintedanib was discontinued 5 days before surgery and resumed after chest tube removal, and no AE‐IPF events or mortality were observed. Most patients had a usual interstitial pneumonia pattern [9]. However, the absence of a control group and the small sample size preclude conclusions regarding the efficacy of nintedanib in preventing postoperative AE‐IPF. In the present meta‐analysis, the included studies similarly focused on patients with IPF, and the available data did not allow us to evaluate the effects of perioperative AFT in patients with non‐IPF interstitial lung diseases, including nonspecific interstitial pneumonia (NSIP). Therefore, the limited evidence for nintedanib cannot be attributed to the prevalence of NSIP on the basis of the available data. The paucity of evidence may be partly attributable to the later approval of nintedanib compared to pirfenidone [8]. Moreover, concerns have been raised regarding potential impairment of wound healing; however, recent studies in lung transplantation have not demonstrated adverse surgical outcomes [28, 29, 30]. Data on safety remain limited. In addition to the mixed retrospective cohort study by Bongiolatti et al. [27], which included both pirfenidone‐ and nintedanib‐treated patients, Sumiya et al. [9] reported no surgery cancellations or postponements due to drug‐related toxicity; the most frequent postoperative complications were prolonged air leakage (25%) and surgical site infections (16.7%). These findings suggest that nintedanib may be feasible in a perioperative setting; however, the small sample size and lack of controls warrant cautious interpretation. Further investigations are warranted to elucidate the efficacy and safety of perioperative nintedanib administration in lung cancer surgery.

Notable regional differences have been reported in the causes of death among patients with IPF. In Asian populations, particularly in Japan and Korea, AE‐IPF and lung cancer are the leading causes of death (AE‐IPF, 40%–46%; lung cancer, 8%–11%), whereas in Western populations, the incidence of AE‐IPF is lower and cardiovascular events are more common [31, 32, 33]. For example, in a US population‐based study by Fernández Pérez et al. [33], ischemic heart disease accounted for approximately 23% of acute dyspnea‐related deaths within 4 weeks, and cardiovascular disease represented a major cause of mortality overall. These epidemiologic distinctions may partly explain why perioperative prevention of AE‐IPF has been actively investigated in Japan but remains less emphasized in Western settings. Moreover, although pirfenidone has been the principal focus of preventive studies in Japan, the limited perioperative use of nintedanib may reflect concerns not only regarding wound‐healing complications but also its potential cardiovascular risk [34]. As the regional patterns of mortality and comorbidities differ, further research in Asian populations may help elucidate the role of nintedanib in the prevention of AE‐IPF and its safety in the perioperative setting.

### 4.1 Clinical Implications

The JACS score, which incorporates the history of AE, surgical procedures, computed tomography findings of usual interstitial pneumonia, male sex, preoperative steroid use, Krebs von den Lungen‐6 (KL‐6) levels, and vital capacity, provides valuable information for stratifying the surgical risk in patients with IPF [6]. In line with this, Kanayama et al. [24] reported that intermediate/high‐risk patients, as defined by the JACS score, experienced a significantly lower incidence of 90‐day AE‐IPF when treated with perioperative pirfenidone. These findings underscore the importance of integrating risk stratification tools when evaluating the preventive effects of AFT in the perioperative setting.

In addition to pharmacological strategies, less invasive approaches such as uniportal video‐assisted thoracoscopic surgery (U‐VATS) may reduce surgical invasiveness compared with multiportal VATS [35, 36, 37, 38]. Although the risk of AE‐IPF is determined by multiple factors beyond the surgical approach [6], the degree of surgical invasiveness may also influence postoperative outcomes. Therefore, in patients at particularly high risk, less invasive surgical approaches may be considered as part of a comprehensive strategy to minimize the risk of postoperative AE‐IPF and mortality.

### 4.2 Strengths and Limitations

The major strength of this study is that, in addition to confirming the preventive effect of AFT on 90‐day outcomes, we demonstrated significant associations with lower rates of both AE‐IPF and mortality within 30 days of lung cancer surgery. This highlights the importance of considering early postoperative outcomes that are particularly relevant to surgical decision‐making. Nevertheless, this study has several limitations. First, all included studies were retrospective observational studies in which treatment allocation was determined according to clinical practice rather than randomization [23, 24, 25, 26, 27]. Consequently, confounding by indication cannot be excluded, as patients receiving perioperative pirfenidone may have differed from control patients with respect to baseline disease severity, pulmonary function, perioperative management, and other unmeasured clinical characteristics that could have influenced postoperative outcomes. Second, four of the five studies were conducted in Japan [23, 24, 25, 26], and only one was from Italy [27], potentially limiting generalizability. Furthermore, several studies used historical control groups. Improvements in surgical techniques, perioperative management, and supportive care over time may also have contributed to the observed associations independently of AFT. Third, Urushiyama et al. [26] used a national inpatient database that included patients with non‐lung cancer and employed a different definition of AE‐IPF; thus, only 30‐day mortality data were used in the sensitivity analysis. Fourth, we were unable to formally assess publication bias due to the limited number of included studies. Therefore, publication bias remains an unquantified but plausible source of overestimation of the observed associations, particularly given the contrast between our observational findings and the null result of the randomized trial. Fifth, the small number of studies included in each meta‐analysis limited the statistical power to detect between‐study heterogeneity. Therefore, the observed *I* ^2^ values of 0% should not be interpreted as evidence of clinical or methodological homogeneity across studies. The limited number of retrospective studies also limits the overall strength of the available evidence. Sixth, the Italian study included patients receiving chronic pirfenidone or nintedanib in addition to prophylactic pirfenidone. Because only one included study contained patients treated with nintedanib, the independent effect of nintedanib could not be evaluated. Seventh, detailed information on the underlying UIP and NSIP patterns was not consistently available across the included studies, precluding assessment of whether the associations with perioperative AFT differed according to disease pattern. Future primary studies should report the proportions of UIP and NSIP patterns to facilitate evaluation of potential differences in treatment effects according to disease pattern. Finally, although three Japanese studies [23, 24, 25] reported JACS scores for preoperative risk assessment, two studies [26, 27] did not.

Preoperative pulmonary function is also an important determinant of postoperative outcomes in patients with IPF undergoing lung cancer surgery. Although pulmonary function data were summarized in Table 2, the reported parameters (e.g., percent predicted forced vital capacity [%FVC], vital capacity [VC], and diffusing capacity of the lungs for carbon monoxide [DLCO]) varied across studies. Therefore, meta‐regression analysis to evaluate the influence of preoperative pulmonary function was not feasible due to the variability in reported parameters and the limited number of included studies. As a result, residual confounding related to baseline pulmonary function cannot be completely excluded when interpreting our findings. Accordingly, the associations observed in this meta‐analysis should not be interpreted as evidence of a causal treatment effect but rather as hypothesis‐generating findings that require confirmation in adequately powered prospective studies and RCTs.

**TABLE 2:** Patient characteristics and outcomes.

Study	No. of eligible patients		Age	Sex (male)	Pulmonary function test	JACS score	Surgical sites	Type of lung resection	Outcomes			
	AFT group	Non‐AFT group							30‐Day AE‐IPF	90‐Day AE‐IPF	30‐Day mortality	90‐Day mortality
Iwata et al. [23]	31	19	68.0 ± 7.7 (36–82) vs. 68.7 ± 10.1 (43–83)^a^	28 (90.3%) vs. 18 (94.7%)	FEV1%: 94.5 ± 17 vs. 102.7 ± 17, DLCO%: 74.3 ± 22 vs. 80.8 ± 21^b^	10.5 ± 2.2 vs. 11.2 ± 1.5^b^	Surgery for lung cancer	Anatomic resection: 23 (74.2%) vs. 18 (94.7%), wedge resection: 8 (25.8%) vs. 1 (5.3%)	0 (0%) vs. 2 (10.5%)	1 (3.2%) vs. 4 (21.1%)	NA	NA
Kanayama et al. [24]	28	72	73.7 ± 5.4 vs. 72.6 ± 7.0^b^	26 (92.9%) vs. 62 (86.1%)	FVC%: 92.7 ± 15.7 vs. 96.6 ± 17.2, FEV1%: 74.1 ± 9.9 vs. 72.4 ± 9.2^b^	10.9 ± 2.6 vs. 9.4 ± 3.5^b^	Surgery for lung cancer	Anatomical resection: 24 (85.7%) vs. 59 (81.9%), wedge resection: 4 (14.3%) vs. 13 (18.1%)	1 (3.6%) vs. 10 (13.9%)	1 (3.6%) vs. 12 (16.7%)	NA	NA
Sekihara et al. [25]	36	20	72 (51–86) vs. 72 (60–81)^c^	32 (88.9%) vs. 18 (90.0%)	VC%: 94 (60–128) vs. 97 (65–132), FEV1%: 79 (62–93) vs. 79 (59–97)^d^	11 (8–14) vs. 11 (10–13)^c^	Surgery for lung cancer	Wedge resection: 3 (8.3%) vs. 2 (10.0%), segmentectomy: 0 (0%) vs. 1 (5.0%), lobectomy: 31 (86.1%) vs. 12 (60.0%), pneumonectomy: 2 (5.6%) vs. 1 (5.0%)	2 (5.6%) vs. 4 (20.0%)	3 (8.3%) vs. 4 (20.0%)	0 (0%) vs. 3 (15.0%)	1 (2.8%) vs. 4 (20.0%)
Urushiyama et al. [26]^e^	121	484	< 60: 10 (8.3%) vs. 32 (6.6%), 60–69: 37 (30.6%) vs. 151 (31.2%), 70–79: 60 (49.6%) vs. 260 (53.7%), ≥ 80: 14 (11.6%) vs. 41 (8.5%)	107 (88.4%) vs. 436 (90.1%)	Not reported	Not reported	Surgery for lung cancer: 91 (75.2%) vs. 369 (76.2%), surgery for other organs: 4 (3.3%) vs. 18 (3.7%)	Not reported	Severe respiratory complication: 3 (2.5%) vs. 41 (8.5%), mechanical ventilation: 2 (1.7%) vs. 28 (5.8%), high‐dose steroid: 2 (1.7%) vs. 13 (2.7%)	NA	2 (1.7%) vs. 16 (3.3%)	NA
Bongiolatti et al. [27]	29 (chronic pirfenidone 9 (31%), chronic nintedanib 5 (17.3%), prophylactic pirfenidone 15 (51.7%))	26	72 (66–77) vs. 75.5 (66–78)^d^	21 (72.4%) vs. 20 (76.9%)	FVC%: 89 (79.3–100) vs. 98 (83.2–107.2), FEV1%: 88 (78–101) vs. 96.5 (83.2–105.5), DLCO%: 58 (45–67) vs. 65.5 (52.7–76)^d^	Not reported	Surgery for lung cancer	Sublobar resection: 15 (51.7%) vs. 11 (42.3%), minimally invasive surgery: 25 (86.2%) vs. 17 (65.4%)	NA	1 (3.4%) vs. 6 (23.1%)	0 (0%) vs. 2 (7.7%)	0 (0%) vs. 3 (11.5%)

Abbreviations: AE, acute exacerbation; AFT, antifibrotic therapy; ALAT, Latin American Thoracic Society; ATS, American Thoracic Society; BMI, body mass index; COPD, chronic obstructive pulmonary disease; DLCO, diffusing capacity of the lung for carbon monoxide; ERS, European Respiratory Society; FVC, forced vital capacity; GAP index, gender, age, physiology index; HRCT, high‐resolution computed tomography; IPF, idiopathic pulmonary fibrosis; JACS, Japanese Association for Chest Surgery; JRS, Japanese Respiratory Society; NA, not available.

^a^ Mean ± standard deviation (range).

^b^ Mean ± standard deviation.

^c^ Median (range).

^d^ Median (interquartile range).

^e^ Data presented for patients after propensity score matching.

## Author Contributions

**Akimasa Sanagawa:** conceptualization, data curation, formal analysis, writing – original draft. **Hideo Kato:** data curation, formal analysis, writing – review and editing. **Hajime Fujimoto:** writing – review and editing, supervision. **Tetsu Kobayashi:** supervision, writing – review and editing. **Takuya Iwamoto:** supervision, writing – review and editing.

## Funding

The authors have nothing to report.

## Disclosure

Generative AI was not used in the preparation of this manuscript.

## Conflicts of Interest

The authors declare no conflicts of interest.

## Supporting information


**Figure S1:** Sensitivity analysis of 30‐day postoperative acute exacerbation of idiopathic pulmonary fibrosis using the Peto odds ratio.
**Figure S2:** Sensitivity analysis of 30‐day mortality using the Peto odds ratio.
**Figure S3:** Sensitivity analysis of 90‐day mortality using the Peto odds ratio.

## Data Availability

The datasets generated and/or analyzed during the current study are available from the corresponding author upon reasonable request.
