# Supplementary material for: Perioperative Antifibrotic Treatment and Early Postoperative Outcomes in Patients With Idiopathic Pulmonary Fibrosis Undergoing Lung Cancer Surgery: A Systematic Review and Meta‐Analysis
Source: Thorac Cancer. 2026 Oct 25;17(20):e70408. doi: 10.1111/1759-7714.70408 (PMC13696746; doi:10.1111/1759-7714.70408)
Supplement: Supplementary file 1 [file TCA-17-e70408-s001.docx]

**Supplementary Figure S1. Sensitivity analysis of 30-day postoperative acute exacerbation of idiopathic pulmonary fibrosis using the Peto odds ratio**.

Forest plot showing the pooled Peto odds ratio for postoperative AE-IPF associated with AFT. AFT: antifibrotic therapy; OR: odds ratio; CI: confidence interval.

**Supplementary Figure S2. Sensitivity analysis of 30-day mortality using the Peto odds ratio.**

Forest plot showing the pooled Peto odds ratio for 30-day mortality associated with AFT. AFT: antifibrotic therapy; OR: odds ratio; CI: confidence interval.

**Supplementary Figure S3. Sensitivity analysis of 90-day mortality using the Peto odds ratio.**

Forest plot showing the pooled Peto odds ratio for 90-day mortality associated with AFT. AFT: antifibrotic therapy; OR: odds ratio; CI: confidence interval.
